# Race/Ethnicity, Vascular Aging, and Mortality Risk: Evidence from the Health and Retirement Study

**DOI:** 10.1093/geroni/igab046.3162

**Published:** 2021-12-17

**Authors:** Kevin Heffernan, Janet Wilmoth, Andrew London

**Affiliations:** Syracuse University, Syracuse, New York, United States

## Abstract

Vascular aging, which is associated with cardiovascular disease risk and mortality, is characterized by increasing arterial stiffness. The gold standard method for the assessment of arterial stiffness is carotid-femoral Pulse Wave Velocity (cfPWV). An emerging body of research suggests that cfPWV can be reasonably estimated from two commonly measured clinical variables—age and blood pressure. Thus, estimated Pulse Wave Velocity (ePWV) holds promise as a novel and easily obtained measure of arterial stiffness that can be used to study vascular aging, particularly with nationally representative datasets that collect biomarker data on sufficiently large sample sizes to examine race/ethnic differences. This analysis uses data from the 2006-2016 Health and Retirement Study to examine race/ethnic variation in the relationship between ePWV and mortality risk. We estimate logistic regression models predicting mortality over an eight-year period for four racial/ethnic groups: White, Black, Other, and Hispanic. Controls are included for sociodemographic characteristics, health status and behaviors, and blood biomarkers such as C-reactive protein, cystatin-C, hemoglobin A1c, total cholesterol and high-density lipoprotein (HDL) cholesterol. The results indicate ePWV increases the risk of mortality in the total sample and among each race/ethnic group, net the effect of age, systolic blood pressure, and diastolic blood pressure. Mechanisms that mediate this relationship are explored. The findings provide insight into vascular aging processes that influence mortality risk among race/ethnic groups.

